# Supplementary material for: A scoping review of trauma informed approaches in acute, crisis, emergency, and residential mental health care
Source: BMC Psychiatry. 2023 Aug 7;23:567. doi: 10.1186/s12888-023-05016-z (PMC10405430; doi:10.1186/s12888-023-05016-z)
Supplement: Supplementary file 4 — Supplementary Material 4 Table of study characteristics and results [file 12888_2023_5016_MOESM4_ESM.docx]

Appendix 4. Table of study characteristics and results

| **Author(s) and year** | **Type of report/ Study design** | **Population (Staff or services user)**  **Sample size** | **Participant characteristics: Age, race, sex, other** | **Service user: Mental health problem/ diagnosis**  **Length of stay:** | **Trauma experiences (N and %)** | **Staff role and length of service** | **Results:**  **Individual outcomes** | **Results:**  **Interpersonal outcomes** | **Results:**  **Service level outcomes** | **Results:**  **System level outcomes** |
| --- | --- | --- | --- | --- | --- | --- | --- | --- | --- | --- |
| The Six Core Strategies | | | | | | | | | | |
| Azeem, Aujla, Rammerth,  Binsfeld, and Jones (2011)  (47) | Cluster-randomised, controlled, cross-over trial | Population: Service users  N=458 records were reviewed | Age:  Mean age of service users who experienced seclusion/ restraint: 14.4 years  Range: 8–17 years  Ethnicity:  Caucasian: 63.3%  African American: 12.6%  Native Americans: 8.9%  Hispanics: 6.3% Other: 8.3%  Sex:  Women: n=276 Men: n=182 | Disruptive behaviour disorders (ADHD, ODD, and conduct disorder): 61%  Other disorders including mood disorders: 52%  Reactive attachment disorder: 24%  Psychotic disorders: 16%  Anxiety disorders: 18%  SUDS: 16%  Pervasive developmental disorders: 14% Intellectual disability: 14%  Length of stay of those who experienced seclusion/ restraint: average: 70.6 days (range 11–177 days). | Not stated | Not stated | Over the study period, n=79 (17.2%) service users (n=44 women, n=35 men) required seclusion and/or restraint.  Of the 79 service users who experienced seclusion and/or restraint, 25 (32%) experienced 3 or more incidents. Service users involved in 3 or more restraint or seclusion incidents had a longer average length of stay in the services (85 days) versus service users involved in fewer than 3 incidents of restraint or seclusion (64 days).  In the six months before the TIC training, the number of restraints and seclusions was 93 (73 seclusions, 20 restraints). These involved 22 service users (n=11 women, n=11 men).  In the six months after TIC training, the number of restraints and seclusions was 31 (6 seclusions, 25 restraints) involving 11 service users (n=7 women, n=4 women).  There were higher rates of disruptive behaviour disorders and mood disorders among service users who experienced seclusions or restraints, versus those who did not experience seclusions or restraints. | None measured | None measured |  |
| Azeem, Reddy, Wudarsky, Carabetta, Gregory & Sarofin (2015)  (48) | Description of service implementation | Population: Service users  Sample size:  2005 – n=178  2014 – n=162 | Service users:  Age:  Not specified, but it is a paediatric hospital,  Race:  2005 (n=178) Caucasian 38%  African American 34%  Hispanic 27%  Other 1%  2014 (n=162), Caucasian 40%, African American 30%  Hispanic 26%  Other 4%.  Sex:  2005 (n=178)  Male: 51%  Female: 49%.  2014 (n=162)  Male: 46%  Female: 54% | Not stated | Not stated | Hospital Executive leaders; including the Superintendent, Assistant Superintendent, Medical Director, Director of Nursing, and Director of Clinical Operations), childcare workers, nurses, clinicians, and physicians. | Mechanical restraints (i.e. restraining service users to restraint beds) decreased by 100% from 485 incidents in 2005 to “zero” in 2014 for 3 years consecutive years in 2012, 2013, and 2014.  Physical restraints, whereby staff physically restrict the service users’ movement, decreased by 88% from 3,033 incidents in 2005 to 379 in 2014. | None measured | None measured | None measured |
| Duxbury (2019)  (57) | Cross-site study, including process evaluation | Population: N/A  Intervention wards (n=144 beds, mean= 20.1 beds per ward)  Control wards (n=147 beds, mean= 21.0 beds per ward) | Participant characteristics: N/A  Primarily mixed gender wards, in the intervention and control wards there were 2 female-only wards and 1 male-only ward in each. | Length of stay: around 15 days | Not stated | Not stated | None measured | None measured | Restraint rates:  Average reduction of physical restraint rates across the intervention wards: 22%.  Rate of restraints was significantly lower on the intervention wards in the adoption phase (6.62 events/1000 bed-days, 95% CI 5.53-7.72) compared to baseline (9.38, 95% CI 8.19-10.55).  There was a temporary increase in restraint rates on the intervention wards during implementation (10.76, 95% CI 9.34-12.19) prior to the decrease observed in the adoption phase.  There were no changes in rates of restraint on the control wards over the same period (baseline: 5.33, 95% CI 4.45-6.20; implementation: 5.65, 95% CI 4.62-6.67; adoption: 7.22, 95% CI 6.01-8.42).  The association between control versus implementation wards, and study phase, was significant (chi-squared=39.15, df=13 p<. 0002) There was a 62% reduction in restraint on implementation wards compared to control wards. | None measured |
| Chandler (2008)  (55) | Qualitative descriptive study design | Population: Staff  N=10 | Not stated | N/A | Not stated | 8 nurses and counsellor providers volunteered to participate, and 2 administrators volunteered.  Day shift staff: n=8  Evening staff: n=1  Night shift: n=1  Length of service: minimum 12 years | Staff reported a gradual change of perspective regarding patient behaviour after learning more about trauma and its effects on service user behaviour. Perspective shifts were reinforced by nurse manager role models, who believed in TIC. | Collaboration was the basis of symptom management.  Staff described that they changed their perspectives, developed collaborative relationships with service users, implemented safety protocols, and used educational resources.  Staff perceived that service users saw others as able to support and help one another to manage their symptoms and cultivate coping skills.  Service users share the community services that work well for them and refer others to successful outpatient programs. Patient networks are key, and now policy has evolved to support patient contact post-discharge, if they want to continue relationships formed in the unit.   There was an emphasis on a less confined physical environment for service users, which has led to units being combined. Service users with substance abuse, mental health, and PTSD are in the same unit, along with older service users and those who are physically unwell, and those who are potentially aggressive. Service users are not separated by diagnosis. Staff have therefore worked to create an environment that encourages relational connections. | None measured | There was a culture shift, moving control from staff to the patient. Creating a structure of active administrative involvement, staff development, skills training, and staffing ratios was required to ensure that staff could implement TIC.  The relationship hierarchy shifted with the introduction of TIC, as they empowered service users to have control over their lives.  Service users and staff have additional opportunities to collaborate on medication and treatment decisions.  An appropriate staffing ratio is key for maintain safety, as well as staff engaging with relevant professional development programmes.  Educational plans that include developing the patient’s knowledge about their symptoms and skills training for coping were developed. |
| Chandler (2012)  (56) | Qualitative case study of one inpatient unit | Population: Staff  N=11 | Not stated  female=7 male= 4 | Not stated | Not stated | Staff roles: Nurses: n=3  Mental health counsellors: n=3  Occupational Therapist: n=1  Occupational Therapist aide: n=1  Administrators: n=3  Years of experience:  Nurses: 2-20 years  Mental health counsellors: 6-25 years  OT: 10 years  OT aide: 2  Administrators: more than 20 years | Staff developed a new perspective on service users’ experiences of PTSD after learning about trauma and its impacts on behaviour.  Staff approached service user behaviours, such as self-injury, in a calm and non-punitive way.  The leadership philosophy that influenced day-to-day staff decisions was to remain interested and engaged with service users’ lives.  Staff and service users were able to develop their trauma-related knowledge and skills, and receive recognition for their engagement.   The nurses, counsellors, and OT reported that they needed to be flexible in order to respond appropriately to service user needs. | Staff were able to raise their safety concerns, and the day-to-day impact of TIC supported them to create a cohesive team.   Developing trusting relationships with patients was a foundational principle of TIC in this setting. Service users feeling understood by staff begins on admission.  Staff-staff relationships were essential to develop treatment plans. The team works with the service users to develop comprehensive treatment plans. | The occupational therapist now performs a sensory assessment with service users to identify individual sensorimotor preferences and to increase service users’ awareness of their physical responses to challenging situations, and to develop personalised plans. | To involve all staff in TIC training, a retreat and a series of compulsory trainings on TIC, were conducted.   Meetings facilitated collaboration between staff and leadership and during each shift this provided the support required for the sensitive, intensive nature of the work to create a secure environment.  Service user groupwork added to the safety plans by providing support for recovery, as they supported service users to identify their treatment goals, and work on their self-soothing practices. |
| Hale (2019)  (62) | Non-randomised controlled trial (this paper reports only on outcomes of physical restraint reduction) | Population: Staff  Sample size: Not stated | Service users: Age: Range 3-17 | Not stated | Not stated | Physicians, nurse practitioners, nurses, behavioural health technicians,  therapists, dietary staff, and ancillary staff who work in admissions and reception. | Crisis interventions before TIC implementation: n=440  Crisis interventions after TIC implementation: n= 259  Seclusions before TIC implementation: n=215  Seclusions after TIC implementation: n=125  Restrains before TIC implementation: n=225  Restraints after TIC implementation: n=134  Use of physical holds and seclusions decreased by 26% within 6 months of TIC implementation. | Staff reported strategies for de-escalation: using their tone of voice, distraction and eye contact.  Staff attitudes: Staff reported a sense of pride knowing they are able to support service users who have experienced trauma, and they reported knowing that TIC has been successful in other settings and believe they can make the same changes in their setting. | Psychiatrists and nurse practitioners, reported less need for documenting crisis interventions that may increase a patient’s length of stay in the service and which may interfere with medication management. | The physical hold and seclusion policy was updated to ensure all crisis interventions were reported. Leadership committed to analysis of restraint and seclusion data to extract trends e.g., the time of day, staff involved.  Staff compared monthly restraint and seclusion data in this setting to others. Seclusion and restraint data was made available to staff and service users, and used to evaluation adoption of the intervention of each floor of the service. Staff were recognised for showing effective de-escalation techniques each month in an ‘all-star’ programme.  Following TIC, yearly teaching for all staff regarding seclusion and restraint was mandatory. Staff must attend an annual refresher that emphasises de-escalation techniques. TIC was featured on all staff job descriptions and yearly evaluation.  Revisions to assessment tools included trauma and aggression histories for completion on admission. During the admission assessment, crisis interventions were introduced to the service users. Each floor had a comfort room, allowing service users in crisis to go to a space that provides a calm environment.  Patient advocates who were on-site five days a week, were introduced. They responded to all concerns and supported the service user and their family. A milieu coordinator was established to act as a buffer between staff and service users.  The debriefing process was improved; all staff involved in the incident were present, learnings from the incident were prioritised, treatment plans were updated, and adherence to procedures were reviewed via body camera footage. Service users were not involved in this process. |
| Hale & Wendler (2020)  (63) | Service change description | Population: Service users  Sample size: N/A | Not stated. | N/A | N/A | N/A | Seclusion: Before TIC implementation: n=215 After TIC implementation: n= 125 Difference: n= -41.86%  Restraint Before implementation: n=225 After Implementation: n= 134 Difference: n= 40.44%  Overall reduction in crisis interventions of holds and seclusions of more than 41%.  Data at one year demonstrated a further reduction of holds and seclusions by 9.3%.  Crisis interventions continued to decrease, with a 4% lessening at 1 year follow-up. | None measured | None measured | None measured |
| The Sanctuary Model | | | | | | | | | | |
| Farragher & Yanosy (2005)  (58) | A quality improvement project | Population: N/A  Residential programme: n=73  Day treatment programme:  N=80 | Not stated | Not stated | Service users:  Exposure to any trauma: 60-70%  Staff:  Exposure to any trauma: 75% | Not stated | Some staff were resistant to creating openness around confrontation styles, as some felt criticised for their previous ways of working. | The Core Team developed a shared language which improved communication between service users and staff and helped them to work through problems and reach solutions.  Staff felt increasing responsibility and began to intervene when they observed a colleague escalating a conflict. | A multidisciplinary team (The Core Team) met regularly, enabling each department to evaluate their strengths, weaknesses, interaction styles and assumptions driving their behaviors and functioning.  Assessment processes were revised to better understand service user needs on arrival.  Collaborative safety planning and including the service user in the process, was prioritised. | None measured |
| Korchmaros, Greene & Murphy (2021)  (67) | Retrospective pre/post study | Population: Staff  Sample size:  Staff: T1 - n= 25 T2 - n= 31 T3 - n=23 | Race:  T1  White 90%  Black 7%  Multiracial 3%  T2  White 91%  Black 6%  Multiracial 3%  T3  White 92%  Black 4%  Multiracial 4%  Hispanic ethnicity:  T1 – 37%  T2 – 34%  T3 – 36%  Sex  Female: T1 – 70%  T2 – 63%  T3 – 60% | Not stated | Not stated | Staff roles: program  directors, regional directors, school principal, clinical supervisors, case managers, therapists, unit coordinators, nurse, overnight staff, and behavioral health associates.  Length of employment:  T2  Employed for at least the past 13 months (therefore including T1) – 61.3%  T3  Employed for  more than 2 years (therefore including T1) – 52.2% | Readiness:  Staff had more negative perceptions of their own implementation readiness (M=2.60) than agency leaders’ readiness (M =3.10) and the agency’s implementation readiness (M = 2.98).  Staff perceptions around agency and agency leader implementation readiness reduced between T1 (M=3.21 and 3.37) and T2 (M=2.98 and 3.02) and remained stable to T3 (M=2.76 and 2.92).  Staff perceptions of their own implementation readiness did not change across time points.   Staff satisfaction: After 2.25 years post TIC implementation (T3), staff were satisfied with Seeking Safety (M=1.57, t(df) 2.73 (22), p<.01) but not The Sanctuary Model (M =1.26), not significant).  Acceptability: At 2.25 years (T3) post TIC implementation, staff were generally accepting of The Sanctuary Model and Seeking Safety and thought that they improved client outcomes.  Staff were likely to adopt intuitive evidence-based TIC practices, were likely to adopt required evidence-based practices, were open to new evidence-based practices, and believed that evidence-based practices were important and useful. This positivity did not change over time. | None measured | Program fidelity: At T3, The Sanctuary Model and Seeking Safety were being used correctly and the implementation of The Sanctuary Model (M 2.57 t(df) 3.21 (22), p <.01) and Seeking Safety (M 2.83 t(df) 5.10 (22), p<0.01) was of good quality.  Feasibility: 4 months (T1) post TIC implementation, staff  perceptions of the feasibility of the implementation of the TI-RSTs were positive.  On average, staff thought that the treatment agency was capable of trauma informed change (M 3.24 t(df) 8.57(24) p<0.01) and sustaining change (M 2.72 t(df) 4.88 (24) p<0.01). Over the next 2 years, these perceptions were stable.   Staff training:  At 1.25 (T2) and 2.25 (T3) years post TIC implementation, most staff reported receiving The Sanctuary Model and Seeking Safety training. At T2 years post TIC implementation, 90% of staff reported receiving any The Sanctuary Model training. At T3 years post TIC implementation, 96% of staff reported receiving Sanctuary Model training.  At T2 years post TIC implementation, 70% of staff reported receiving Seeking Safety training. At T3 years into TIC implementation, 74% of staff reported receiving Seeking Safety training. | None measured |
| Kramer (2016)  (68) | Service description | Population: Service users and staff  Staff:  Focus groups n=10 Interviews n= 3  Service user:  Focus groups n=13 | Not stated | Not stated | Not stated | One focus group consisted of youth care workers with a representation of new hires (experience of one year or less) and experienced workers (between 3 and 5 years’ experience). The second group consisted of clinical staff. | Staff and service users reported an ability to perceive linkages between rights and responsibilities, an increased ability to identify and confront re-enactment situations.  Staff and service users reported an increased ability to articulate realistic goals for themselves while creating strategies for change. They also reported increased hope for the future.  Service users reported that keeping their feelings “in check” is important to reduce the impact on themselves and others.   Service users become active participants in their recovery and contribute to a safety plan and engage with conflict resolution.  There were narrative descriptions of reductions in absconsion, restraint, removal from the programme.   The residents felt empowered and able to trust in others enough to narrate their stories in individual, family, and group therapies and within the Sanctuary milieu. | Service users have found that there is less victim-blaming, punitive, and judgmental responses post TIC. They experienced clearer and consistent interpersonal boundaries, and expected more from their interactions.  Service users reflected on the importance of positive staff support (both formal and informal) in their therapeutic relationships.   Open communication was key, but was still a work in progress as some staff members were not employing communication skills effectively or appropriately. | Service users experienced the setting as a safety net, where there was a sense of community. Service users made use of the community, including by participating in group meetings.  Service users wanted more democratic processes, and staff spoke of developing a service user ‘government’ to facilitate voicing their opinions and concerns.  Sanctuary culture is creating a safe, therapeutic community. The Sanctuary safety planning, psychoeducation, community meeting strategies make the community operate as a therapeutic environment.   Staff who do not buy-in to Sanctuary may be less to committed to delivering the programme and to comply with its strategies and may believe that confounding variables explain the changes observed. | None measured |
| Prchal (2005)  (70) | Service evaluation | Population: Staff  N = 67 | Demographic data was not collected from the study participants.  However, demographic data was available for the service user population.  Age:  Range: 12-20 years  Mean=15 years  Race:  Hispanic 34%  Black non-Hispanic 51%  White 11%,  Asian, Pacific Islander, or biracial 4%  Sex:  Male: 73%  Female: 27% | Not stated, but service was designed for children and young people with ‘conduct disorders and other serious emotional disturbances’ | Service users:  Experienced at least one physical abuse incident: 34%  Experienced at least one sexual abuse incident: 12%  Experienced at least one neglect incident: 45% | Milieu staff members, clinicians (Master's level therapists), administrators | Staff experiences, attitudes and beliefs:  Clinical staff mostly perceived the Sanctuary Model as positive, though some felt it was too abstract. The information given to staff on trauma supported them to be more psychologically informed and to understand service user behaviours with more compassion. This led to less verbal abuse used by the milieu staff, who are subjected to the service users’ aggression most often. Staff overall felt they made and better clinical decisions. Clinical staff reported that the trauma informed nature of the Sanctuary Model was an improvement over previous approaches used in the unit.   The most beneficial components of the Sanctuary Model for staff were the principles that improved safety on the units, encouraged respect and communication among staff and between staff and youth, and encouraged staff to think about and frame negative service user behaviour within a trauma informed framework.  Service user outcomes:  They worked effectively in groups, and leaders emerged. The service users also began to express distress in a less destructive way. | Staff now take the time to explain decisions that directly affect service users, which previously were handed down without explanation, sometimes leading to conflict.  Staff encourage service users to be actively involved in their communities.  Staff communicate more effectively, and more often, with each other. However, a barrier to implementation was the communication between staff in different roles (psychiatrists, therapists and milieu staff), where staff reported feeling dismissed and undervalued.  Staff are now models for respectful behaviour between each other and towards the service users.  Sanctuary language was incorporated into daily communications between staff and youth, creating a shared understanding. | Clinicians increased the time they spent onsite supporting milieu staff.  Service users are given opportunities to develop their own community rules, and to call on their community when an issue is identified.  Service users were also given a key role in developing their personal safety plans. All staff were invited to contribute to each service user’s treatment. | The unit did not create many opportunities or incentives for senior staff to flatten the hierarchy experienced by staff in various roles on the unit.  Implementing the Sanctuary model was resource intensive, and required an entire philosophical change, as well as increased staff training and technical assistance. High staff turnover, and limited administration support, were key barriers to implementation. |
| Rivard, McCorkle, Duncan, Pasquale, Bloom & Abramovitz (2004)  (71) | Pre/post implementation of intervention pilot study | Population: Staff  Ten focus groups; 3 involved clinicians and administrators (n=7–12 in each group), and 7 focus groups involved milieu counsellors (n=3–10 in each group). | Not stated | Not stated | Not stated | Clinicians, administrators/supervisors, milieu counsellors | Staff shifted from thinking that they were responding to bad behaviours from service users, but instead thinking that these were opportunities to teach more adaptive coping mechanisms. | Safety plans encouraged service users to be responsible for their treatment, and rather than depending on program rules, staff supported service users to process their feelings, make decisions with the service users, and to discuss alternatives.  When working with families where there is a trauma history, it is difficult to acknowledge the issues at hand without the families feeling blamed and increasing their guilt and remorse.   Staff are aware that their communication skills are related to safety. The more they communicate, the physically safer the environment is for everyone. Communication helps to build trust and a feeling of closeness. When the environment is more psychologically and socially secure, staff are able to share their ideas, opinions, frustrations, and mistakes more openly with others. | Consistency was promoted by staff training, community meetings, structure time for team building, making the use of the psychoeducation tools a procedure, staff openness, service users listening, maintaining satisfaction and motivation, enthusiasm and constant reinforcement, supporting service users to gain a broader understanding of what it means to recover from trauma, cohesion among staff and service users, providing incentives for positive behaviours in the community, strong program leadership, the presence of staff on the units, and clarity over staff authority figures. Staff felt that TIC helped to make change happen faster.   Barriers to implementation included insufficient time to communicate and do the required team building. There were different ways of managing crises across the units, leading to inconsistency. Not all staff had received the formal TIC training. There was a perceived lack of, or shifting, administrative support and resource allocation, affecting TIC implementation. There were also constraints as a result of the research design which limited the implementation of TIC. Staff reported the need for additional training with more experiential content.  Staff reported that the quality of team meetings and case conferences improved in terms of active involvement staff communication. | None measured |
| Rivard, Bloom, McCorkle, Abramovitz, (2005)  (72) | Service evaluation | Population: Service users and staff  Youth: N=158  Staff: not stated | Service users:  Age:  Range: 12-20 years  Mean: 15 years  Ethnicity:  Hispanic = 33%  Black = 47%  White = 13%  Asian, Oriental, or Pacific Islander = 1%  Bi-racial or other = 6%  Sex:  Men: 63%  Women: 37% | Service users averaged 6 prior placements, and 3 psychiatric hospitalisations | Service users:  At least one incident of physical abuse: 33%  At least one incident of sexual abuse: 14%  At least one incident of neglect (48%)  Seen someone else attacked with a weapon: 42%  Been attacked with a weapon: 23%  Seen someone else shot: 20%  Been shot at: 11% | Not stated | Between baseline and 6 months, the incendiary communication/ tension management scale of the Youth Coping Index scale measures, significantly decreased over time. Scores for the Standard Residential Services significantly increased (p < 0.05).  Scores on the Nowicki-Strickland Locus of Control Scale decreased over time, and they became more internalising, indicating an increased sense of control over their lives. Scores of service users in the Standard Residential Services remained constant (p=0.15).  On the verbal aggression scale of the Social Problem-Solving Questionnaire, scores of service users in the Sanctuary Model decreased slightly, whereas service user scores in the Standard Residential Services increased (p=0.15). | Staff stated that trauma theories were helpful in emphasising the way that issues and behaviours of service users are linked to their experiences and helped them to redirect towards recovery.  Counsellors learned new ways to problem-solve with staff and service users, and these were important to them. | Therapeutic community outcomes were measured by COPES. No significant differences were found across the conditions at baseline and over  the following 2 two phases of measurement. By the final measurement phase, there were significant group differences on the following constructs of the COPES: support (p<0.05), spontaneity (p<0.01), autonomy (p<0.05), personal problem orientation (p<0.05), safety (p<0.05), and in the total score (p=0.001). | Across the units that implemented the model, scores on the Sanctuary Project Implementation Milestones criteria ranged from 66% to 92% (mean=78%).  The most challenging component to implement was the weekly psychoeducation group. Improved implementation was observed in units that had been exposed longer to TIC, and where the leadership demonstrated enthusiasm and commitment to TIC.  Important principles and concepts that staff identified included the central focus on safety and the feeling of community and greater teamwork. |
| Comprehensive tailored trauma-informed model | | | | | | | | | | |
| Boel-Studt & Shamra Marie (2017)  (51) | Service evaluation | Population: Service user  Sample Size:  Total: n=205  Children and adolescents who received traditional Psychiatric Residential Treatment (PRT; n = 100)  Children and adolescents who received Trauma Informed-Psychiatric Residential Treatment (TI-PRT; n = 105, with complete treatment records) | Service user:  Age:  Range: 5-17 years (M=10.53, SD= 2.68).  Race:  White (69.3%) Multiracial (13.7%)  Black (11.2%)  Sex:  Male: 58%  Female: 42%   TI-PRT group (n=105) female n=45, 42.9% PRT group (n=100) female n=41, 41% | Minimum length of stay was 5 weeks. | TI-PRT group (n=105)  Emotional abuse n=60, 57.1% Neglect n=55, 52.4% Physical abuse n=52, 49.5% Sexual abuse n=39, 37.5% Domestic violence n=31, 29.5%  PRT group (n=100) Emotional abuse n=53, 53% Neglect n=45, 45% Physical abuse n=49, 49% Sexual abuse n=30, 30% Domestic violence n=23, 23% | N/A | As Child and Adolescent Functional Assessment Scale (CAFAS) scores increased, the odds of experiencing no restraints decreased (odds ratio=0.98, p=0.007)  Service users receiving TI-PRT spent fewer months in treatment on average (M=6.45, SD=3.07) compared to those in the traditional PRT (M=10.78, SD=4.71).  After controlling for age, sex, race, and CAFAS scores at intake, there was a significant association between treatment condition and length of stay (F(5, 204)=13.07, p=0.000, R^2^ =0.247).  Receiving TI-PRT accounted for a quarter of the variance in length of stay between groups.  Approximately 93% of service users in TI-PRT were discharged to community-based placements compared with 82% in the PRT group, but this was not statistically significant.  CAFAS scores at discharge indicated that service users in both groups improved over the course of treatment. The TI-PRT group experienced greater improvements. | None measured | The number of physical restraints for the sample ranged from 0 to 130. About 37.1% of youth experienced no restraints; Seclusion incidents ranged from 0 to 128 with 64.9% of the sample having experienced no incidents; The average number of restraints in TI-PRT facilities (M = 9.13, SD= 17.38; Median = 3) was higher compared with traditional PRT (M = 6.13, SD = 6.13; Median = 1); Conversely, the average number of seclusion room placements in traditional PRT (M = 10.90, SD = 25.96; Median = 0) was higher than TIPRT (M = 4.19, SD = 9.82, Median = 0) ; The expected number of seclusion room incidents for youth in the TI-PRT condition was less compared with youth in the traditional PRT condition (OR = .41, p = .000); | None measured |
| Brown, McCauley, Navalta, & Saxe (2013)  (53) | Service evaluation | Population: Service users  Boston IRTP: Sample size not stated  KVC Health Systems: n=70 | Age:  All three programs were aimed at children. | Not stated | BOSTON IRTP: Not stated  KVC Health Systems: Service users were exposed to an average of four traumas —the most common being physical abuse, domestic violence, sexual abuse, and exposure to community violence. | Not stated | BOSTON IRTP: There was a dramatic and sustained decrease in physical restraints following TIC implementation. There was no increase in staff or child assaults.   KVC Health Systems: There was a decrease in CAFAS measured functional impairment in all domains, with an average exit score of 56 one year after TIC, compared to an average exit score of 120 for the pre-TIC 2008 comparison group.  For service users discharged, placement stability was 89% to a foster home or kinship placement, compared to placement stability of only 48% for the pre-TIC 2008 comparison group.  Per service user, there was an average of 1.4 placement moves, compared to 3.4 placement moves for a pre-TIC 2008 comparison group.  There was a decrease in the use of both seclusions and restraints in the service. There was a “safety hold” rate of 7.59 per 1000 patient days in May 2010 compared to 37.55 in July 2009. | None measured | None measured | None measured |
| Cadiz et al. (2005)  (54) | Pre-post study | Population: Service user  Sample size:  Not stated, 417 bed facility | Average age: 34  Race/ Ethnicity:  62% African American  8% White, 29.4% Other 41% Hispanic, 24.6% Puerto Rican  Sex:  100% female  Number of children:  85% < 1 child Average of 3 children  Child welfare involvement:  36% of children prior to treatment entry  64.2% of children were staying with family/ others.  Housing: 22% homeless/ living in shelters  Incarceration history: 60% history of incarceration and mandated to treatment | 27% attempted suicide  85% reported mental health difficulties.  100% addiction history  65% were daily drug users | Service users  73% had a trauma history  30% reported childhood sexual or physical abuse  25% reported domestic violence victimisation | Not stated | Service user experiences of TIC: By engaging women in a familiar, safe environment, they were more receptive to the services and the staff.  Group members appreciated the self-determination, in making their own rules, starting the check-in at the start of every group, and validating their experiences. | Cultural considerations: Staff considered the cultural importance of food, and mealtimes, which enhanced the quality of the care, made inter-cultural communication more effective, and assisted the engagement process.  Service user experiences: Through the introduction of TIC, the women built trust with other members of staff and felt able to seek their support. | Not measured | Women’s Treatment Specialist co-led group interventions with mental health staff at the intervention site, which taught existing staff about TIC so that they could take over group leadership, enabled more flexible leadership, and gave service users more options for staff support.   Confidentiality was an issue, which led to some service users opting not to participate. |
| Forrest, Gervais, Lord, Sposato, Martin, Beserra & Spinazzola (2018)  (59) | Pre-post study | Population: Service users  Sample size: N/A | Service users:  Age: Range: 12-22 years | Program 1:  Service users experiencing mood, anxiety, or trauma-related  Symptomatology  Program 2: Service users experiencing mild-moderate  cognitive impairment or developmental delays, and concurrent behavioural  or mental health problems | N/A | N/A | Length of stay:  Programme 1:  2012 (M=19.94 months)  2013 (M=12.15) prior to TIC implementation.  From 2013 to 2017 (M=12.85) length of stay remained stable but was decreasing overall.  Program 2:  Length of stay fluctuated in the 4 years before TIC implementation. Length of stay decreased from 2015-2016 (M=24.84 months), then increased in 2017 (M=28.80 months).  Restraint use:  Program 1:  Staff utilized an average of 112.63 restraints per quarter pre-TIC implementation. Restraint use fluctuated throughout the study period. Post-BCC implementation an average of 68.80 restraints was utilized per quarter.  Program 2:  At the onset of TIC implementation, there was variable use of restraints. Staff used on average 62.47 restraints per quarter. Restraints were high prior to TIC implementation (n=117) but this reduced post-implementation (n=18). Overall, there was a small reduction in the use of restraints post TIC implementation. | None measured |  | None measured |
| Goetz & Taylor-Trujillo (2012)  (60) | Description of service implementation, and pre/post intervention study | Population:Staff and service users  Sample size: N/A | Not stated | N/A | N/A | The team that planned and introduced the TIC model included of the director of clinical services, the director of risk management,  the employee health nurse, and the clinical program  manager. | Prior to TIC implementation, staff injuries in 2006: n=103  During year 1 of TIC intervention, injuries were reduced by 48%.  In 2006, the hospital census increased with a large number of new employees in the organization with limited relevant experience. This was thought to contribute to the level of injury. Staff were also adjusting to the introduction of the PFI model.  Seclusion and restraint rates were reduced by at least  50%. The duration of seclusions and restraints reduced by 75% over the first 2 years after TIC implementation.  Patient satisfaction scores and staff safety survey data reflected  a culture change and a change in treatment of patients occurred in all areas of the facility.  In 2008, 1 year after the implementation of TIC, the staff safety survey showed improvements in 50% of the areas, including how staff perceived the effectiveness of the patient aggression management program.  There was a decrease in the total number of patient events (Code Gray events), and in the number of mechanical restraints episodes since the introduction of TIC. | None measured | None measured | None measured |
| Stamatopoulou, (2019)  (73) | Mixed methods case study | Population: Staff  N=20 | Age:  Range: 24-62 years  Ethnicity:  White British n=18 (90%), Multiracial n=1 (5%)  Asian British n=1 (5%)  Sex:  Female: n=20 (100%) | N/A | Service users:  N=29/31 of the service users endorsed traumatic experiences on the HCR-20 V3 risk assessment.  94% of service users from December 2017 reported experiencing one or more traumatic event(s). | A multi-disciplinary team which comprises nursing, psychiatry, psychology, occupational therapy, dietetics, speech and language therapy, social work and physiotherapy.  Nursing background n=18 (90%)  Psychotherapists n=2 (10%)  Length of service:  Range: 3-20 years | Staff engaged in a process of unlearning their personal attitudes towards service users and themselves as professionals after TIC implementation. Staff also reflected that these attitudes may have impacted way they viewed the transition to TIC and added to the fear and anxiety within the team.  They reconnected with the job satisfaction which some had lost. Staff reported feeling valued and felt a sense of achievement having implemented TIC. The culture of working in a forensic unit may have resulted in internalised attitudes of thinking that you needed to remain stoic. There was an evolving appreciation and focus on staff welfare, which was supported by increasing awareness around people’s boundaries and recognising their personal experiences of adversity and trauma, and of vicarious trauma.  Staff were about what was expected from them when TIC was implemented, as it was a new way of working, potentially leading to low confidence and a need for reassurance. The shift was experienced as a sudden change. A stepped approach may have been less overwhelming. Staff reported that their uncertainty lessened as they began to see the benefits of TIC.  Staff reported increased empathy towards service users after training, and felt they had more in common with service users than they initially thought.  Staff also felt surer of what they had to offer service users, and more able to respond to situations of escalating risk. | There was a connectedness between the staff, and between the staff and the service users. Staff talked about the increasing time and space shared with service users, and this led to a culture of shared learnings. This happened through group reflections and the introduction of recovery processes after incidents.  Staff established openness through trust and experience/information sharing in the team. The transition to TIC introduced staff to a new paradigm of understanding distress which moves away from the medical model. By adopting a trauma focused lens, participants could learn about service users and make links between service users’ backgrounds and their behaviour. Staff were then much more empathetic towards service users. | Within the third theme: "navigating new practices" Discovering new ways to manage risk has been one of the practices that participants seem to directly link to the reduction of incidents in the unit while there seems to be a deep appreciation for the introduction of structure in the working day. | Staff felt that TIC introduced significant uncertainty within the team about their practice. Regular meetings with staff were important to ensure consistent delivery of TIC. New staff, who were untrained, posed a risk to TIC consistency across the setting, which could lead to miscommunications. Training new starters was recognised as a key barrier to implementation, as there is limited time and money for services to be implemented and developed.  Staff reported that since the implementation of TIC, there was an increase in the platforms available for talking through the impact of the work including reflective practices, debrief sessions and handovers. This reflects the organisational structures for practical support required by the TIC model.  Open information sharing altered staff practice and enabled staff to support service users. Systems for recording information were not in line with the TIC model requirements, which were to prioritise information about service user trauma and to make it accessible. This could pose a threat to the consistent delivery of the TIC model since it could prevent staff teams from accessing important information about service users. |
| Tompkins & Neale (2016)  (74) | Cross-sectional correlational study | Population:Service users and staff  Staff: N=18  Service users: N=19  Among service users: current clients: n=9  former clients who had completed treatment: n=5  former clients who had left the service prematurely n=5 | Staff: Not stated  Service users:  Age:  Range: 25-44 years.  Ethnicity:  White British n=16  Other n=3.  Sex:  Women n= 9 (100%).  Previously engaged within sex work: n=7  Previously incarcerated: n=4 | History of substance use issues ranging from 2 to 25 years  n=7 were in treatment for Class A drug use  n=7 were in treatment for alcohol use  n=5 were in treatment for combined Class A drug and alcohol use.  Length of stay: Initial funding for stays of 12 weeks, additional funding available to stay for an additional 12 week | Not stated | Staff roles:  Key stakeholders who had established the TIC service n=3 Current TIC staff n=7  Former TIC staff n= 3, staff working elsewhere in the organisation n = 5.  Length of service: Range: between 4 months and 17 years  Mean length of service the current TIC service: 32 months. | Service users:  Service users felt that the structured daily routine (including a consistent treatment timetable) added to their feelings of safety and contrasted with the unpredictability of their day-to-day life.  Service users reported being unaware that the service was guided by a trauma-informed approach and did not know what the treatment involved before arriving. They were not expecting to have to learn about trauma, discuss their emotions, foster therapeutic alliances, or reflect on their emotional and behavioural history.  All staff described strategies for maintaining their wellbeing, including not working overtime and taking sufficient breaks. Staff reported practising mindfulness and attending mutual aid groups to protect their own emotional welfare.  Staff described feeling initially unclear about what TIC entailed and felt they were learning as they went from their colleagues. Working together and establishing monthly mentoring and supervision supported staff to develop confidence and skills to deliver TIC and to reflect on their practice.  Staff:  Staff reported practising mindfulness or attending groups to maintain their recovery and protect their own emotional health.  TIC staff needed to maintain their personal and professional boundaries to protect their own well-being and approach service users consistently.  A challenge of TIC was staff avoiding becoming too emotionally or personally involved with service users, and feeling impacted by service users’ experiences. This could potentially undermine their ability to deliver TIC.   Participation in treatment at times unsettled service users, such as when they were new to the service, or when having difficulties with their emotional regulation. Due to the unpredictable nature of the setting, staff were conscious of the volatility of the environment and were ready to react quicky when necessary. | Service users’ previous traumatic experiences and their difficulties opening up and sharing their experiences undermined their ability to trust, impacting their bonds with staff. They struggled to trust staff, be open, or cultivate relationships; service users acted defensively or vigilantly as a self-protection mechanism.  Service users who left the service before completion reported not engaging with the programme or cultivating therapeutic alliances with staff. This was because they did not want, or did not feel able, to confront their traumatic experiences.  Staff and service users felt that therapeutic relationships were at the heart of how TIC worked. Service users felt most understood by staff members who had experienced addiction. Working collaboratively with service users to understand their needs was key.   Staff stated that supporting service users in a trauma-informed way required them to be available, flexible, consistent, nurturing, and honest. However, staff recognised that they should not be overprotective of service users.  Staff felt it was easier to develop therapeutic relationships with women who were fully detoxed, used to attending treatment groups, determined to stop using substances, and curious about exploring their trauma experiences alongside their addiction. These service users were more stable overall, had fewer immediate needs, and were more ready to engage with treatment.   Central to feeling secure in the service was getting on with other women, developing relationships, and being in a homely atmosphere. | Staff felt that safety was promoted by organisational policies, such as preventing service users from having visitors. They also felt that the daily routines and activities supported a feeling of stability.  Staff reported that service user safety was promoted when trauma-informed treatment groups were held in large spaces. Staff advocated the use familiar rooms which were women-only.   The organisation commissioned a two-day intensive TIC staff training. Staff reported that the bespoke training supported them to deliver the manualised TIC programme, facilitate groups, take service users trauma histories into account, and learn how to support service users’ trauma responses.  Despite the training, staff still reported that it had took time to feel competent and confident in using TIC. Staff employed after the training had been delivered were also not trained. | TIC requires a high ratio of staff to service users, as significant staff attention and support is required.  At recruitment, it was highlighted that it is important to consider each candidate’s experience and background, and how that complemented the needs of the service users. Managerial staff believed that staff should be women, and have personal qualities, experiences, and skills that would support them to work effectively with the service users. They recruited women who had their own experiences of addiction recovery and of working in this field previously.  Stakeholders and staff noted that they would not work in the service indefinitely and that time off would be required if unwell. Treatment quality might be affected if staff were absent, due to needing to take time off. During staff shortages, current staff workloads, which affected the quality of their work and increased their stress and feelings of burnout. |
| Zweben et al. (2015)  (75) | Service report and evaluation | Population: Service users and staff  Total: n = 95  Intervention group: n=44  Non-intervention group: n=51 | Sex:  Women: 100% | Alcohol misuse: 100%  Average length of stay was 206 days in the intervention group, compared to 128 days in the non-intervention group. | Not stated | N/A | Fewer participants in the intervention group reported psychological or emotional problems in the last 30 days (n=21, 41%), compared to entry to the programme (n=40, 78%)  Service users reported reduced drug or alcohol use in the past 30 days (43% on entry compared to 6% after six months).  Average length of stay was 206 days in the intervention group, compared to 128 days in the non-intervention group.  Reunifications with children approached 100%, as Family court and protective services learned of the service users’ recovery. | Staff reported that the trauma-informed program led some families to realise that other family members also needed mental health and/or substance abuse treatment, and many of these family members have been served by other EBCRP programs. | None measured | None measured |
| Blair et al. (2017)  (50) | Pre-post study | Population: Service user  Baseline n=3884  Post intervention implementation n=8029 | Service users:  Age:  Baseline:  4.9 % < 12 years 85.9 % > 13–65 9.2 % > 66  Study sample:  5.0 % < 12 years 87.2 % > 13–65  7.8 % > 66  Race:  Baseline:  15.9 % Black  23.9 % Spanish/ Hispanic  56.3 % White  3.9 % Other  Study sample: 16.5 % Black  3.6 % Spanish/ Hispanic  55.3 % White  4.6 % Other  Sex: Baseline:  49.7 % female 50.3 % male.  Study sample: 48.5 % female 51.5 % male | Not stated | Not stated | N/A | Rate of seclusions decreased by 52% (p<0.01):  Study period 213/8029 = 4.4/100 admissions vs. baseline 358/3884 = 9.2/100 admissions).  Rates of restraints decreased by 6 % (non-significant, p=0.44): baseline 213/3884 = 5.5/100 admissions, study period 412/8029 = 5.1/100 admissions.   Duration of seclusions per admission during the study period reduced by 27%.  Duration of restraints per admission increased by 52%.  Mean seclusion duration exceeded mean restraint duration during baseline (337.7 vs. 286.0 min, p=0.02) and during the study period (516.2 vs. 445.0 min, p = 0.27).   Comparing baseline to the study period, seclusion rates reduced, and the mean seclusion duration increased (337.7 to 516.2 min, p<0.01). From baseline to post-intervention the mean duration of restraints increased from 286.0 to 445.0 min (p<0.01). | None reported | Of events which were formally reviewed (190 of 411 events, 50.6 % of which were seclusion and 49.4 % restraint events) the mean BVC score was 2.58 (categorised as ‘‘high risk’’).  In 99.3 % (n = 120) the patient had a history of positive response to comfort/sensory interventions | None reported |
| Safety focussed tailored trauma-informed models | | | | | | | | | | |
| Borckardt et al. (2011)  (52) | Qualitative, data included: group observation; content analysis of agency documents and quantitative data; focus groups; and individual interviews | Population: Service users and staff  Sample size: Service user Quality of Care questionnaires: n=446  Staff Quality of Care questionnaires = 340 | Not stated | Not stated | Not stated | Not stated | Baseline restraint rate (mean±SD):  0.027±0.018 per patient day  Follow up restraint rate (mean±SD):  005±0.002 per patient day.  The TIC model was associated with an 82.3% reduction in use of seclusion and restraint during baseline and follow-up (Wilcoxon=6.00, z=–2.65, p=0.008).  The implementation of change to the physical, therapeutic environment was associated with a significant reduction in seclusion and restraint (F=7.94, df=1 and 119, p=.006).   Perceptions of the therapeutic environment (M±SD):  Staff:  Baseline: 3.5±0.41  Follow up: 3.78±0.29  Service users:  Baseline: 3.72±0.16  Follow up: 3.94±0.18  (t=2.07, df=8, p= .04).  Rating of patient involvement in treatment planning (M±SD):  Baseline: 3.75±0.32  Follow up: 3.92±0.48  Staff trauma sensitivity rating (M±SD):  Baseline: 4.25±0.10  Follow up: 4.30±0.08  Trauma sensitivity rating (M±SD):  Baseline: 4.25±0.13  Follow up: 4.22±0.12   Patient perceptions of the physical environment (M±SD):  Baseline: 3.72±0.16  Follow up: 3.94±0.18  (t=2.07, df=8, p= .04).  Patient perceptions of their involvement in treatment planning (M±SD):  Baseline: 3.88±.15  Follow up: 4.08±.12  (t=2.33, df=8, p=.02) | Patients’ ratings of the trauma sensitivity of the staff (mean±SD):  Baseline: 3.88±0.23  Follow up: 3.97±0.25  Ratings of trauma sensitivity (mean±SD):  Baseline: 3.85±0.23  Follow up: 3.92±0.21 | None measured | None measured |
| Jones (2021)  (66) | Pre-post study | Service users and staff  Service user: n=23 (57% of the patient population)  Staff: n=13 (34% of the nurse population) | Age:  Service users: M = 38.6  Range = 19-55  Staff:  M = 43  Sex:  Service users: Women: n = 23 (100%)  Staff:  Women: n =11, Men n = 2 | The service users are detained under the Mental Health Act, have mental disorders, and have been assessed as presenting an immediate risk to others.  Mean length of service use: 6.6 months Range: 6 months - 20 years. | Not stated | Staff role: Named nurses  Length of service:  Mean employment: 9 years  Range: 1-36 years. | The submissive position service users experience when in distress was described as reflective of roles enacted within past abusive relationships. Feeling lonely was characterised by many of the service users as being separated from family. The service users felt separate from their families and felt this was poorly addressed by the service. Service users described a fear of being discharged back into the communities where they had previously experienced significant personal traumas.  Service users had greater insight into how their behaviours affect the people around them, and their work with named nurses increases their independent recognition of their distress and how to cope.  Women reported feeling safer and more contained in the current TIC approach than previously.  Staff acknowledged feeling insufficiently prepared for dealing with self-harm and they felt anger at the system for placing them in these difficult situations. Staff stated insufficient supervision was a key reason for nurses experiencing work-related post traumatic symptoms. Their coping strategies included being kind to and praising themselves for getting through the difficult moments.  Nurses accepted the psychological traumas experienced at work, and expected to overcome these struggles alone and for them to continue nursing unaffected. | Service users described remorse and shame following aggressive behaviours towards staff, which led to distress and further aggression. Distress was felt by both service users and staff. Service users emphasised the physicality of their distress, but staff described the interventions as mostly cognitive or emotional. The nurses reflected that the intervention insufficiently met the service users’ need for acquiring sensory awareness and self-soothing skills.  The lack of appropriate language and difficulty articulating distress was highlighted, and so the development of a distress vocabulary was recommended.  Difficult therapeutic relationships left some service users feeling rejected and uncared for, while several nurses felt rejected and victimised.  Involving the service users in staff training was reported by both staff and service users as beneficial in the development of a shared understanding and for enacting cultural change. Shared understanding was key in nurturing therapeutic relationships. Shared understanding reduced barriers that limited the service users’ articulation. Therapeutic relationships were experienced as intensely emotional. Nurses wanted to connect with the service users over their shared victimisation experiences, and their inability to do this was felt as a barrier to building connections, and perpetuated power imbalances. Gaining knowledge of the service users was easier if the service users felt that the process of gaining knowledge was a joint venture.  Nurses experienced work traumas associated with patient behaviours, so it was important for nurses to see the service users as vulnerable to harm, to encourage the provision of compassionate care. When service users behaved aggressively, some nurses were more critical of their colleagues’ lack of compassion.   Engagement improved when nurses offered their time, as this discredited the service user belief that nurses were too busy to support them. | Sensory interventions were identified as a key need of the service users, and so were adopted by the NHSHSW. Training in sensory interventions was incorporated as standard practice.  The nurses reported that they needed specialist training, ongoing support and supervision to appropriately support service users.  Lack of time was reported to be most damaging to the therapeutic relationship, and the nurses felt that much was expected from them in terms of the containment of the service users and the support of colleagues.  Service users perceived the staff training to be key in developing the motivation and ability to be compassionate, which goes beyond knowledge to cover interpersonal proficiency. | Nurses feeling under pressure from the amount of work they had to complete, more so than the pressure felt as a result of the service users’ distress. The pressures of the job were described as depriving the service users of the nurses’ time. |
| Trauma informed training intervention | | | | | | | | | | |
| Aremu, Hill, McNeal, Petersen, Swanberg, Delaney (2018)  (46) | Qualitative focus groups | Population: Staff  Total: N=32  Initial training wave: n= 11  Second wave of training: n=22 | Staff:  Age (years):  Initial training wave: M=41.7, SD=12.2  Second training wave: M=45.95, SD=15.56  Sex:  Initial training wave:  Women: n=6 Men: n=5 Second training wave:  Women: n=21  Men: n=1  Initial training: Associate’s degree: n=7 (63.6%). Second training wave: High school: n=7, (31.8%)  Associate’s degree: n=6 (27.3%)  College: n=3 (13.6%)  Baccalaureate in nursing: n=5 (22.7%) Masters: n=1 (4.5%) | N/A | Not stated | Initial training wave:  Nurses: n=5  Behavioural Health Technicians (BHTs): n=6   Second training wave:  Nurses: n=14  BHTs: n=8  Length of service:  Initial training wave: Years working in psychiatry (mean): 7.3 years (SD=7.4).  Second training wave:  Years working in psychiatry (mean): 8.19 years (SD=10.96). | Initial training wave: PRE/post Combined Assessment of Psychiatric Environments (CAPE):  Pre-test score: 32.4 (SD=5.3)  Post-test score: 33.9 (SD = 5.9)  No statistical difference (p=0.087)  Pre/post scores MAVAS scale: Pre-test score: 66.5 (SD=6.6) Post-test score: 69.8 (SD = 5.6) No statistical difference (p=0.244)  Second wave of training: PRE/post on the CAPE:  Pre-test score: 31.6 (SD = 3.2)  Post-test score: 32.6 (SD = 4.9) No statistical difference (p=0.323)  Pre/post scores MAVAS scale: Pre-test score: 70.2 (SD = 11.9)  Post-test score: 69.2 (SD = 10.1) Statistically significant difference (p=0.010) | None measured | None measured | 2015: Number of PRN intra-muscularly (IM) medications administered per week: 11.9  2017: Number of PRN IM medications administered per week: 12.1  Following the second wave of training, PRN IM medications administered per week decreased.  April: n=3.2 per week  May: N=5.4 per week |
| Gonshak (2011)  (61) | Qualitative | Population: Service users and staff  Service users: n=92  Staff: n=7 | Age (years)  Service users: Mean 16.2  Range 11-18  Staff:  Range: 28-61  Ethnicity:  Service users: Caucasian: 67%  African American: 31%  Hispanic: 2%  Staff: Caucasian: n=5  African American: n=1  Other: n=1  Sex:  Service users: 100% female Staff:  100% female | Many service users reportedly had emotional and behaviour disabilities, mild intellectual disability, and specific learning disability. | Not stated | Not stated | Staff:  Trauma informed beliefs (mean, SD):  Pre-training: 3.3 (0.38)  Post-training: 3.4 (0.44).  Risking Connections Knowledge (mean, SD):  Pre-training: 0.52 (0.20)  Post-training: 0.55 (0.24)  Service users:  No statistically significant differences in reported trauma symptomology pre to post TIC training (t=0.58, p=0.56) or in their perception of the student-teacher relationship (t=-0.83, p=.41). | CLASS - Emotional support (mean, SD):  Pre-training: 5.0 (1.26)  Post-training: 4.8 (0.91) | Staff:  Risking Connections Fidelity (mean, SD):  Pre-training: 2.1 (0.57)  Post-training: 2.1 (0.38) | None measured |
| Niimura, Nakanishi, Okumura, Kawano & Nishida (2019)  (69) | Service evaluation | Population: Staff  N = 65 | Age (years):  Mean: 42.2  SD: 11.9  Sex:  Women n=56 (86%)  Men n=9 (14%) | N/A | Not stated | Registered nurse n=53 (82%)  Occupational therapist n=5 (7.7%)  Psychiatric social worker n=3 (4.6%)  Practical nurse n=2 (3.1%)  Other n=2 (3.1%)  Length of service in current role:  Mean: 16.2 years  SD: 11.3  Length of service in psychiatric field  Mean: 7.8 years  SD: 7.0 | Mean score of the Attitude Related Trauma-Informed Care scale scores significantly from 5.1 during pre-TIC training to 5.5 after TIC training (mean difference: 0.4; 95% CI 0.3–0.5) and 5.4 three months later (mean difference: 0.3; 95% CI 0.2–0.4). 48% reported that they had implemented TIC practices after training (n= 27/56).  The most frequently reported clinical practice was ‘assessing patients’ behaviour based on TIC’ (37%). 50% of participants felt that they had ‘limited skills and/or experience of TIC’ and lacked confidence to implement TIC.  86% of staff who did not implement TIC into their practice reported barriers to doing so, such as a lack of time (reported by 40%), limited skills and/or confidence (reported by 24%) and lack of opportunities to implement TIC in their current role (reported by 40%). | Modifying communication with patients based on TIC and managing patients’ behaviours without being coercive was implemented by 19% of staff.  Staff paid attention to their tone of voice, volume, and/or the choice of words when communicating with service users.  n=7 (44%) staff members reported difficulties sharing information regarding TIC with colleagues, while three others attempted sharing TIC training, but their colleagues were not interested. | None measured | None measured |
| Other trauma informed models | | | | | | | | | | |
| Beckett, Holmes, Phipps, Patton & Molloy (2017)  (49) | Non-randomised control design (with nested process evaluation) | Population: N/A  27-bed ward,  divided into a six-bed high dependency  unit and 21-bed acute unit. | Not stated. | Average length of stay: 11 days  Monthly separation rate: >70 service users. | Not stated | N/A | After de-escalation and safety training, combined with training on trauma-informed perspectives, nursing staff’s confidence increased and they felt motivated to engage therapeutically with service users who were extremely distressed.  Nurses exhibited a greater awareness of childhood and adult adversity, and this led to greater understanding, compassion, and respect for service users.  After three years, seclusion rates reduced by 80% and use of security staff was minimised. The duration of seclusion incidents mostly reduced to <60 minute. | There was reduced use of clinical jargon and derogatory descriptions of service users, and staff increased their efforts to focus on service user strengths during clinicial discussions or handovers. | Management ensured regular opportunities for informal staff and service user groups to meet to discuss their concerns, experiences, and ideas for improvement. Admission information was improved to make the process more transparent.  A sexual safety training module was developed and attended by all staff, leading to revised ward policy and procedure.  Therapeutic groups were improved, and a broader therapeutic range of services were provided.  A staff working group investigated the use of medication in the unit and conducted a literature review to identify best practice, leading to revisions of ward protocols on rapid sedation. | None measured |
| Isobel & Edwards (2017)  (64) | Qualitative evaluation of implementation of a TIC intervention | Population:  Staff  n=5 | Not stated (due to small sample size) | N/A | Not stated | Mental health nurses  Length of service: Range: 1 – 25+ years | All nurses hoped hopeful that TIC would improve nursing care, making it more consistent and cohesive while also more individualised and flexible.  Staff agreed that change needed to be slow and positively framed to be accepted and implemented successfully. TIC wasn’t always fully understood, and some nurses felt that the introduction of TIC was a criticism of their current approaches, which were being labelled as traumatising. Nurses reported wanting clarity, consistency, and clear role expectations within TIC.  Some nurses questioned whether the trauma informed changes were significant. Others feared that a lack of safety could arise from changes to long standing practise. | None measured | None measured | None measured |
| Jacobowitz, Moran, Best, & Mensah (2015)  (65) | In-depth case study approach | Population: Staff  N=172 (n=158 had complete information) | Age (n=169) < 21 0.6% 21–30 36.1% 31–40 18.3% 41–50 20.7% 51+ 24.3%  Race (n=169)  African American 29.6 % Asian 0.6 % Caribbean 13.6%  Pacific Islander 1.2%  Latino 6.55 % White 42  Male 33.1% Female 66.9%  Last Trauma Informed Care Meeting (n=160) Last week 25.0% Last month 19.4% Last 3 months 16.3% Last 6 months 21.9% Last 12 months 9.4% 12+ months 8.1%  Education (n=172):  High school 13.4% 1–3 years of college 43.6% Bachelors 27.3% Masters 13.4% Doctorate 2.3% | N/A | Staff (n=160):  Verbal Assault Last month: M 4.4  SD 6.8  Last 6 months  M 15.7  SD 25.5 Lifetime  M 18.3  SD 17.0  Verbal threat Last month M 1.5  SD 2.8 Last 6 months  M 3.4  SD 6.1 Lifetime  M 13.7  SD 16.8  Physical attack Last month  M 0.8  SD 1.8 Last 6 months  M 2.0  SD 3.4 Lifetime  M 10.0  SD 13.7  Severe physical attack Last month  M 0.3  SD 1.0 Last 6 months  M 0.8  SD 1.8 Lifetime  M 1.9  SD 3.2 | Inpatient psychiatric health care workers, that consisted of registered nurses (RNs), psychiatric aides (unlicensed paraprofessionals that assist the RNs), assistant counsellors (group therapists), psychiatrists, case coordinators (discharge planners), and therapeutic rehabilitation specialists (psychiatric rehabilitation).  Years of experience: <1, 7% 1-2, 20.3% 3-4, 22.7% 5+ 49.4% | Post-traumatic stress symptoms increase as the length of time between attending trauma-informed care meetings increases (β=0.021, t=2.16, p<0.03), and signs of burnout increase (β=0.014, t=4.12, p<0.00).   A model predicting post-traumatic stress symptoms (PTSS) among staff included the total frequency of severe physical attacks, staff age, TIC meeting attendance, and compassion fatigue. This model explained 23.4% of the variance with respect to PTSS. There was a significant correlation between TIC meeting attendance and adjusted PCL-C scores (r = -0.156 p<0.05) | None measured | None measured | None measured |
| Prytherch, Cooke & Marsh (2020)  (28) | Pre-post study | Service users  N=8 | Age:  Range: 22-53 years  Race:  White British n=5, British Chinese n=1  Black African n=1  Greek Cypriot n=1  Sex:  Women: n=8 (100%) | Length of stay: Women stayed two weeks in the crisis house on average, the maximum stay was four weeks.  n=7 service users had been admitted to hospital within the last four years.  n=1 had been admitted nine years previously. | Not stated |  | Service users acknowledged that the medical-custodial approach experienced in hospital was effective in keeping service users alive, but wasn’t emotionally supporting them long-term. The trauma informed crisis house prioritised the cultivation of therapeutic relationships and permanent emotional healing.   Participants reported that it was difficult to be directly asked about trauma. However, it became easier subsequently to name and discuss their experience of trauma, though talking had limits. One participant did not identify as a trauma survivor, and did not feel deserving of her distress.  Although the service users felt cared for, TIC was mainly focused on individual support and failed to address wider societal injustices that contribute to distress.  Service users reported experiencing more freedom, privacy and comfort in the crisis house compared to in hospital.  Having a safety plan which enabled service users to leave the house, supported them to maintain social roles such as employment, which helped to avoid institutionalisation. | Service users described that the formation of trusting relationships was key in developing strategies to stay safe. Service users felt able to discuss their feelings with staff, to hand blades in, or to ask for support.  All service users valued the regular one-to-one meetings with consistent staff members in the crisis house. The women reported feeling safer in a women-only environment, and it made it easier to be open. When give sufficient time and individual attention to open up and bond, the service users felt valued and were able to be honest. Feeling cared for and also being involved in their care decisions enabled a trust to develop with staff.  Trusting relationships were described as healing for service users. | N/A | N/A |
